# Autism-like behaviors and abnormality of glucose metabolism in offspring derived from aging males with epigenetically modified sperm

**DOI:** 10.18632/aging.104061

**Published:** 2020-10-13

**Authors:** Wen-Long Zhao, Ni-Hao Gu, Zheng-Zheng Li, Gui-Shuan Wang, C. Yan Cheng, Fei Sun

**Affiliations:** 1 The International Peace Maternity and Child Health Hospital, School of Medicine, Shanghai Jiao Tong University, Shanghai, China; 2 Shanghai Key Laboratory of Embryo Original Diseases, Shanghai, China; 3 Shanghai Municipal Key Clinical Speciality, Shanghai, China; 4 Institute of Reproductive Medicine, School of Medicine, Nantong University, Nantong, Jiangsu, China; 5 The Mary M. Wohlford Laboratory for Male Contraceptive Research, Center for Biomedical Research, Population Council, New York, NY 10065, USA

**Keywords:** advanced paternal age, glucose metabolism, autism-like behavior, intergenerational and transgenerational offspring, DNA methylation

## Abstract

Accumulating evidence from epidemiological studies of humans and genetic models in rodents has shown that offspring from males of advanced paternal age (APA) are susceptible to metabolic and neurological disorders. However, knowledge of molecular mechanism(s) underlying these metabolic and behavioral changes at the intergeneration and trans-generation levels from APA is limited. Here, we characterized changes on glucose and cholesterol metabolism, and also autism spectrum disorders (ASD)-like behaviors in 1^st^ and 2^nd^ generations from 12- and 18-month-old male mice, respectively. Whole Genome Bisulfite Sequencing (WGBS) of sperm from APA mice identified differentially methylated regions (DMRs) within the whole genome, and DMRs within promoter regions, suggesting that specific genes and relevant pathways might be associated with autism and aberrant glucose metabolism in the offspring from APA males. These results strongly suggest that epigenetic reprogramming induced by aging in male sperm may lead to high risks of aberrant glucose metabolism and the development of ASD behaviors in intergenerational and transgenerational offspring.

## INTRODUCTION

The paternal role on conception and subsequent metabolic programming and neurodevelopmental alterations of offspring is an emerging concern among married couples and supported by medical evidence [1]. It is increasingly clear for a strong association between advanced paternal age (APA) and psychiatric disorders as well as aging-related pathology among offspring of aging couples [2–4]. However, some of these findings, in particular those based on psychiatric analysis and sexually dimorphic phenotypes remain controversial. This is due to, at least in part, differential aged human population and mice used in analyses and/or studies, and also the use of different analyzing time points between male and female offspring. On the other hand, the metabolic features and autism-like behavioral traits, and the underlying mechanisms of APA on these two parameters, have not been carefully evaluated among intergenerational and transgenerational offspring.

To address these questions, 2-month-old females were intercrossed with males aged 2 months (hereafter, young father, YF), 12 months (hereafter as old father, OF) and 18 months (hereafter very old father, VOF), corresponding to the human age 20s/30s, 40 [5] and 50s/60s, respectively [5], to obtain their F1 offspring, namely, YF-F1, OF-F1 and VOF-F1. F2 offspring were obtained from F1 offspring which were then subjected to cross-breeding and divided into five groups as follows: 1) YF-mF1 x YF-fF1; 2) OF-mF1 x YF-fF1; 3) VOF-mF1 x YF-fF1; 4) OF-mF1 x OF-fF1; and 5) VOF-mF1 x VOF-fF1. Using these mice, we determined whether APA affected the glucose and lipid metabolism and behavioral traits of autism spectrum disorders (ASD) in the F1 and F2 offspring. We next analyzed the DNA methylation status in F0 sperm to identify the possible epigenetic mechanisms of the detrimental effects in the offspring from old fathers. We provided data that aberrant glucose metabolism rather than lipid metabolism was emerged at the juvenile stage and progressed well into adult age in OF-F1 and VOF-F1 mice. The severity of metabolic dysregulation was increased with the advancing paternal age, and associated with sexually dimorphic features. Moreover, the OF-F1 and VOF-F1 male, but not female, mice displayed the core symptoms of ASD. These abnormalities in metabolism and psychiatry were accompanied by substantial epigenetic changes in the aged mice sperm. Furthermore, we reported here for the first time that aberrant glucose metabolism and core symptom of ASD behaviors were transmitted to F2 offspring from OF-fF1 X OF-mF1 and VOF-fF1 X VOF-mF1, while the abnormal phenotypes were less evident than those detected in F1 old father offspring.

## RESULTS

### No significant alterations in glucose metabolism and ASD-like behaviors in OF-F0 and VOF-F0 mice

**Figure 1:**
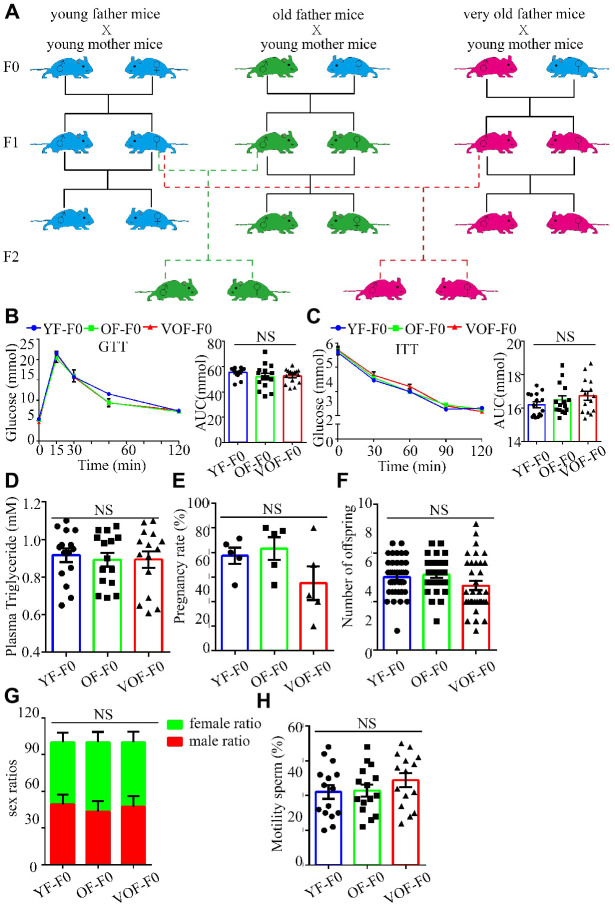
**Experimental design and establishment of advance paternal age (APA) mouse models.** (**A**) Experimental design to obtain F1 and F2 offspring mice. Blue, green and red cartoon mice indicate young father mice (2 months old, YF-F0), old father mice (12 months old, OF-F0) and very old father mice (18 months old, VOF-F0) and their progenies. (**B**) Blood glucose during the glucose tolerance test (GTT) (n=15/group for each group) (left panel) and area under the curves (AUC) results (right panel). (**C**) Blood glucose during the insulin tolerance test (0.5 U/kg) (n=15/group for each group) (left panel) and AUC results (right panel). (**D**) The plasma triglyceride in three groups. (n=15 for each group). (**E**–**H**) The pregnancy rate (n=5 for each group) (**E**), litter size (n=40, 31, 35 litters, respectively) (**F**), sex ratio (n=5 for each group) (**G**) and sperm motility (n=15 for each group) (**H**). Data are presented as the mean ± s.e.m. NS, P≥0.05, versus control (linear regression analysis for GTT assay and one-way ANOVA for other assays).

We first measured blood glucose during the glucose tolerance test (GTT) and insulin tolerance test (ITT) to select OF founder mice with normal glucose metabolism status to rule out the founder effect. In fact, we found most of OF-F0 male and VOF-F0 male mice performed normally in glucose metabolism in the GTT and ITT (Figure 1A–1C), whereas the body weights increased in both OF mice groups (data not shown) that might owe to insufficient exercise and aging traits of old mice in the laboratory. Moreover, OF-F0 and VOF-F0 mice did not alter the levels of plasma triglyceride levels, pregnancy rates, litter sizes, sex ratios, and sperm motility (Figure 1D–1H).

### Higher risk of aberrant glucose metabolism, not lipid metabolism in OF-F1 and VOF-F1 male mice

**Figure 2:**
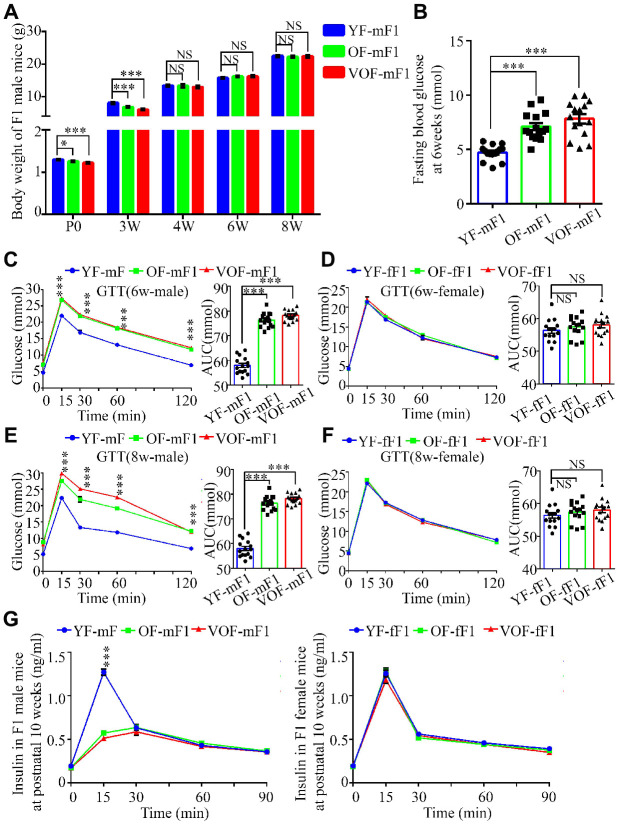
**Reduced body weight, impairment of glucose tolerance and insulin secretion in OF-F1 and VOF-F1 male, not female, offspring.** (**A**) The body weight trajectories in male F1 offspring (n=30 male F1 offspring for each group, ≥ 5 litter size/group). (**B**) Fasting blood glucose. (n=15 male F1 offspring for each group, ≥ 5 litter size/group). (**C**–**F**) Blood glucose during glucose tolerance test (left panel) and AUC results (right panel) at 6 (**C** and **D**) and 8 (**E** and **F**) weeks of age (n=15/each sexual offspring, ≥ 5 litter size/group). (**G**) Plasma insulin concentration in glucose-stimulated conditions. (n=15/each sexual offspring, ≥ 5 litter size/group). YF-mF1, OF-mF1 and VOF1-mF1 were referred as to the F1 male offspring from young, old and very old F0 father, respectively, meanwhile YF-fF1, OF-fF1 and VOF1-fF1 were referred as to the F1 female offspring from young, old and very old F0 father. Data are presented as the mean ± s.e.m. NS, P≥0.05; *, P<0.05, ***, P<0.001, versus control l (linear regression analysis for GTT and insulin assays and one-way ANOVA for AUC results).

APA reduced the body weight in both OF-F1 and VOF-F1 male offspring at postnatal day 0 and 3 weeks of age in comparison to YF-F1 male offspring, but not in female offspring (Figure 2A; Supplementary Figure 1A). However, the body weights of both OF-F1 and VOF-F1 male offspring reached normal level at 4, 6 and 8 weeks of age (Figure 2A; Supplementary Figure 1A). Next, we measured the blood glucose at fasting and during GTT and ITT in F1 offspring at 6 and 8 weeks of age. The male OF-F1 and VOF-F1 offspring displayed higher blood glucose and increased blood glucose rise during GTT (Figure 2B, 2C). It was noted that their response during the ITT was impaired at 6 weeks of age (Supplementary Figure 1B), although the AUCs of male OF-F1 (OF-mF1) and VOF-F1 (VOF-mF1) were higher compared to that in male YF-F1 (YF-mF1). A similar alteration was observed at 8 weeks, and the impairment of GTT in VOF-mF1 offspring was found to be more apparent than that of OF-mF1 (Figure 2C, 2E). Furthermore, we also measured GTT and ITT on some mice at 20, 30 and additional weeks and these findings are consistent with the data from 6 and 8 wk (data not shown). No significant impairments were observed in any female F1 offspring (YF-fF1, OF-fF1 and VOF-fF1) (Figure 2D, 2F; Supplementary Figure 1C). Furthermore, plasma insulin concentrations after glucose stimulation in OF-F1 and VOF-F1 males but not in female offspring, were reduced at 10 weeks, in comparison to that in YF-F1 (Figure 2G). Moreover, the levels of plasma triglyceride were not altered in any F1 offspring from OF-F0 and VOF-F0 male mice when compared to that of YF-F0 male mice (Supplementary Figure 1D).

### Abnormalities in glucose metabolism were transmitted to the F2 offspring mice from old father males intercrossed with F1 female offspring from old father mice

**Figure 3:**
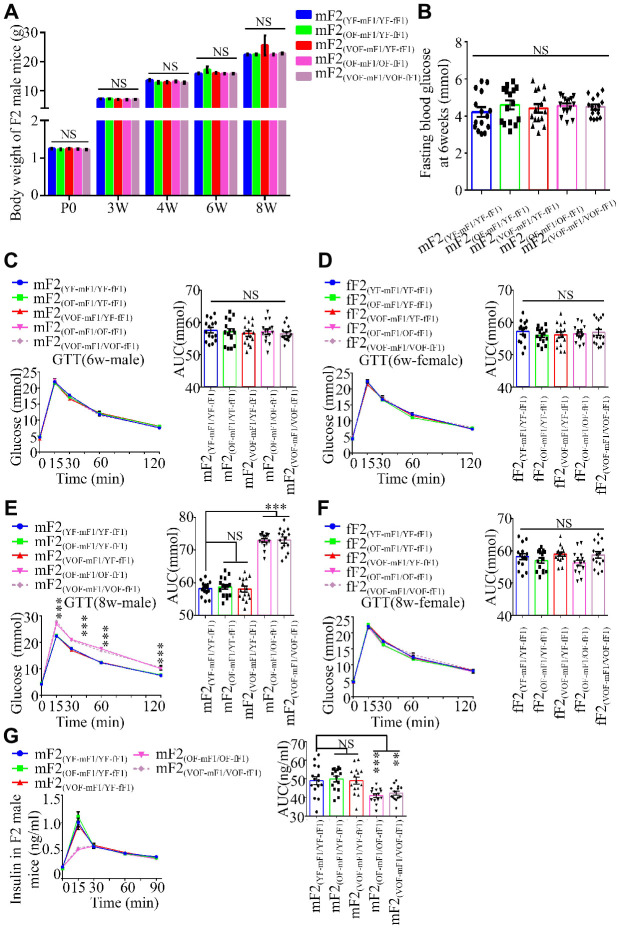
**Body weight, blood glucose during GTT and insulin concentrations in F2 progeny.** (**A**) the body weight trajectories in male F2 progeny (n=30 male offspring for each group, ≥ 5 litter size/group). (**B**) Fasting blood glucose. (n=15 male offspring for each group, ≥ 5 litter size/group). (**C**–**F**) Blood glucose during GTT at 6 (**C** and **D**) and 8 (**E** and **F**) weeks of age (n=15/each sexual offspring, ≥ 5 litter size/group). (**G**) Plasma insulin concentration in glucose-stimulated conditions (n=15/male offspring for each group, ≥ 5 litter size/group). mF2 and fF2 were referred to the male and female progenies from F1 mice, respectively. Data are presented as the mean ± s.e.m. NS, P≥0.05; ***, P<0.001, versus control (linear regression analysis for GTT assay and one-way ANOVA for the body weight trajectories, fasting blood glucose and AUC results).

Next, we investigated if the abnormalities in glucose metabolism were inheritable to F2 offspring mice. Thus, body weight trajectory, fasting blood glucose and blood glucose during GTT (Figure 3A–3F) and ITT as well as the insulin concentrations in F2 male and female offspring were examined (Figure 3 and Supplementary Figure 2). The blood glucose levels during GTT, rather than other parameters noted above, were found to be considerably abnormal in the male offspring from OF-mF1 x OF-fF1 and VOF-mF1 x VOF-fF1 at 8 weeks (Figure 3B and 3E), when compared to other male offspring. Furthermore, the insulin concentrations in mF2 (OF-mF1 x OF-fF1) and mF2 (VOF-mF1 x VOF-fF1) were aberrant (Figure 3G). However, we did not find any abnormalities in glucose metabolism in all F2 female offspring from all five groups (Figure 3D, 3F and Supplementary Figure 2). These results suggested that the APA might still alter the epigenetic status in their female F1 offspring, even though female offspring did not exhibit any significant alteration in glucose metabolism. Furthermore, these adverse effects in F1 male offspring from APA mice mating with female offspring from APA mice were capable of transmitting to male F2 offspring.

### Increased risk of ASD-like behaviors was observed in OF-F1 and VOF-F1 male mice and their F2 progeny when mated with female offspring from APA mice

**Figure 4:**
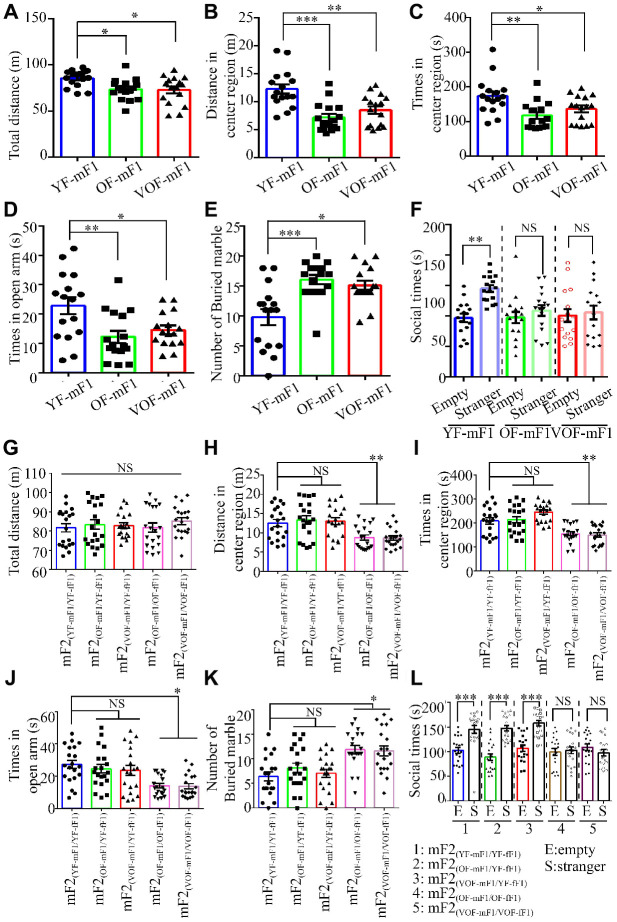
**Autism spectrum disorder (ASD)-like behaviors in F1 and F2 male offspring from old and very old father mice.** (**A**, **G**). The total distance in the open field (OF) in F1 male offspring, *n*=16 mice/group (**A**) and F2 male offspring, *n*=20 mice/group (**G**). (**B**, **H**). The distance in the central area of the OF in F1 male offspring, *n*=16 mice/group (**B**) and F2 male offspring, *n*=20 mice/group (**H**). (**C**, **I**). The time spent in the central area of the OF in F1 male offspring, *n*=16 mice/group (**C**) and F2 male offspring (**I**). (**D**, **J**) The time spent in the open area of the elevated plus maze in F1 male offspring, *n*=16 mice/group (**D**) and F2 male offspring, *n*=20 mice/group (**J**). (**E**, **K**) The number of buried marbles in F1 male offspring, *n*=16 mice/group (**E**) and F2 male offspring, *n*=20 mice/group (**K**). (**F**, **L**) The social time for three chamber tests in F1 male offspring, *n*=16 mice/group (**F**) and F2 male offspring, *n*=20 mice/group (**L**). Data are presented as the mean ± s.e.m. NS, P≥0.05; *, P<0.05; **, P<0.01; ***, P<0.001, versus control (unpaired two-tail t-test for three chamber assay and one-way ANOVA for other behavioral assays).

Earlier studies suggest that APA is a high risk contributing factor of ASD in the offspring from mice and human [6, 7]. Thus, we examined whether the offspring from OF-F1 and VOF-F1 and relevant F2 offspring exhibited ASD-like behaviors in our model. First, we found that both the locomotor activity and the time spent in the central area of the open field in the OF-F1 and VOF-F1 were significantly reduced than that in the YF-F1 (Figure 4A–4C). Also, the time spent in the open arms of the elevated plus maze in OF-F1 and VOF-F1 was less than in YF-F1 (Figure 4D). Using the marble-burying assay to assess the repetitive and perseverative behaviors, the OF-F1 and VOF-F1 performed enhanced marble burying when compared to YF-F1 (Figure 4E). In three chamber assays, the OF-F1 and VOF-F1 displayed social deficits compared to YF-F1 (Figure 4F). Furthermore, we analyzed the behavior paradigm in their F2 offspring. Similarly, the male offspring from OF-mF1 x OF-fF1 and VOF-mF1 x VOF-fF1 displayed ASD-like behaviors compared to other F2 male offspring, consistent with earlier findings [8]. Taken together, these data suggest increased ASD-like behaviors in both OF-mF1 and VOF-mF1 and the male offspring from OF-mF1 x OF-fF1 and VOF-mF𒙂 x VOF-fF1. These data also suggest that the F1 female offspring might increase the susceptibility to ASD in F2 male progeny from OF and VOF-F1 male mice.

### DNA methylation analyses identified methylation alterations in glucose metabolism-associated and autism-associated genes in F0 sperm

**Figure 5:**
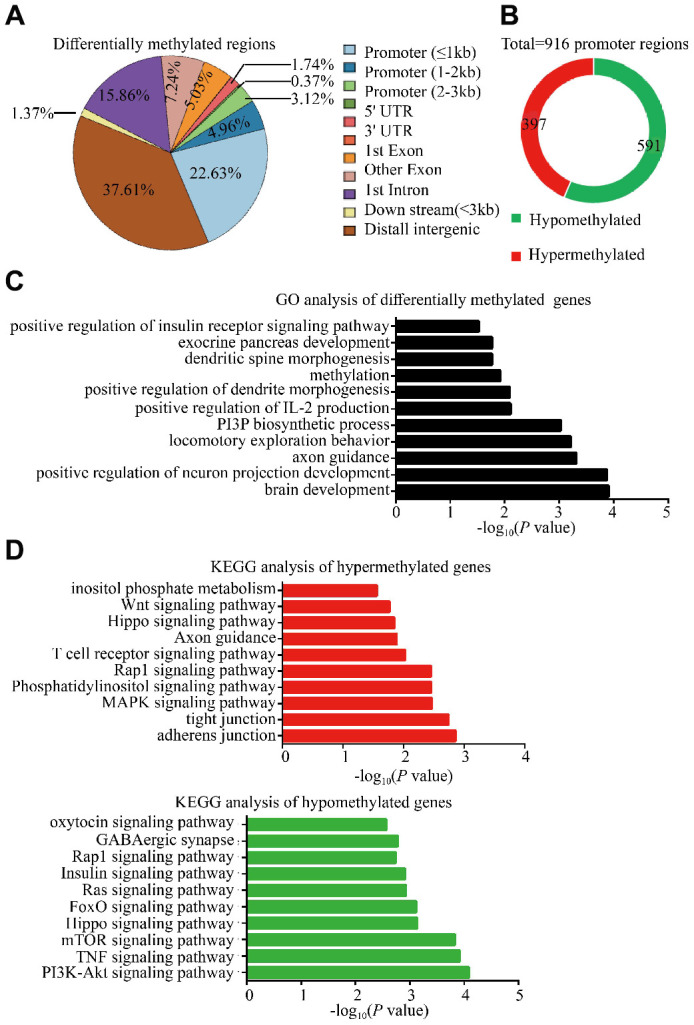
**Methylation changes in F0 aged sperm implicated alterations in several autism-associated and glucose metabolism associated pathways.** (**A**) Whole genome bisulfite sequencing (WGBS) identified differentially methylated regions (DMRs) in F0 aged sperm (*n*=2), showing the distribution of DMRs (q<0.05) in comparison to F0 young sperm (*n*=3). (**B**) the pie plot showed the total number of DMRs within promoter regions. Red and green indicate hypermethylated and hypomethylated promoter regions in F0 aged sperm, respectively. (**C**) Gene ontology (GO) analysis of biological function identified significantly enriched terms in DMRs within whole genome. (**D**) KEGG analysis revealed significantly enriched pathways among the genes with DMRs in whole genome. Red and green bars indicate the hypermethylated and hypomethylated regions in F0 aged sperm, respectively.

Accumulating evidence indicates that epigenetic alterations in germ line cells contribute to the inter-and/or transgenerational phenotypes in metabolic and neurodevelopmental processes, with DNA methylation being a critical and most notable epigenetic modification associated with many cellular processes [9–11]. In particular, changes in DNA methylation in parental genome during embryonic development and altered DNA methylation in gametal genome may play a role in autism and metabolisms [12, 13]. Therefore, the DNA methylation alterations in sperm of two F0 18-month-old male mice and three young male mice, were performed by WGBS to identify potential mediators related to intergenerational and transgenerational inheritance of the observed phenotypes. These results exhibited widespread methylation alterations in whole genome level including 2984 Diff CpG, 20 Diff CHG and 26 Diff CHH, of which the DMR were in the promoter regions (30.71%) and distal intergenic regions (37.61%) (Figure 5A; Supplementary Tables 1 and 2) in DNA. The DMR in the promoter regions were thought to affect gene expression, and we thus further analyzed the methylation status of all promoter regions and the GO and KEGG pathways of the relative DMR genes. There were 988 promoter methylation regions which were found to have been altered, 591 of which were significantly hypomethylated (~59.82%) and 397 significantly hypermethylated (~40.18%) in sperm of old males (Figure 5B). GO enrichment analysis of DMRs in whole genome showed that the biological functions of DMR genes were enriched in insulin receptor signaling pathway, brain development and immunity system response (Figure 5C; Supplementary Table 3). KEGG pathway analysis revealed several pathways with relevance to autism and glucose metabolism, which included mTOR signaling, TNF signaling, oxytocin signaling pathway and insulin signaling pathways in hypomethylated regions; whereas the phosphatidylinositol signaling, MAPK signaling, Hippo signaling and Wnt signaling in hypermethylated regions in aged sperm (Figure 5D; Supplementary Table 4). Taking collectively, these analyses have identified authentic DNA methylation changes in aged sperm, which might contribute to the aberrant development of diabetes and ASD-like behaviors in OF-F1 and F2 mice.

## DISCUSSION

Although alterations in metabolic features and behaviors in APA mice and grand-paternal aged mice models have been previously reported [8, 14], the current study has provided insightful information that reveals APA not only leads to impaired glucose metabolism, but also destructive social ability in F1 male mice. More important, these traits were transmitted and exacerbated to F2 male mice under specific epigenetic background, namely from OF-F1 parental mice. Moreover, our study ruled out the disparities in the glucose metabolism, behaviors and reproductive traits between APA and young founders to minimize the founder effects. Thus, the findings considerably expand earlier studies that APA is associated with aberrant metabolism and ASD-like behaviors based on epidemiological studies and animal models. It is also noted that the observed phenotypes in the mice models reported here do not fully resemble pervious results. For example, adverse lipid profiles noted in the present study are inconsistent with an earlier report which may be attributed to the different ages of mice model (the current very old mice is 18 month old vs >21 months old in an earlier report) [15]. Regarding autism like behaviors, the F1 older offspring from APA mice in another report showed no differences in locomotor activity and anxiety [14], which is inconsistent with the anxiety performance in F1 offspring of APA in the results reported here and an earlier study [8]. This discrepancy may also be due to the different ages of mice used for the corresponding studies (less than 12 months in an earlier report [15] *vs*. 12 months and 18 months in the present study, and 15 months in an earlier work [8]). Nonetheless, these results provide a clue that paternal aging would cause more adverse effects on metabolism features and/or behavioral paradigm in the next generation. Of note, the epidemiological studies in autism documented the odds ratio is increased from 1.64 to 9.39 along with father’s age from 30 to 59 [7].

Furthermore, it is believed that changes in DNA methylation are critical for the occurrence of autism [16] and dysregulation of glucose metabolism, and the DNA methylation pattern in sperm may be inheritable into offspring [17]. In the present study, WGBS analyses have demonstrated the methylation changes in whole genome of aged sperm. GO and KEGG analyses in differentially methylated whole genome regions (include promoter regions) provided the potential pathways associated with aberrant glucose metabolism and ASD-like behaviors, indicating the potential molecular mechanisms underlying the intergenerational and transgenerational effects. For example, it was shown here that hypermethylation at the *Igf2* (insulin like growth factor 2) promoter in aged sperm (Supplementary Figure 3A). Previous reports have shown that down-regulation of *Igf2* leads to insufficient insulin production and secretion and then dysregulation of glucose metabolism [18]. Interestingly, Igf2 protein can reverse the core autism symptoms in the BTBR mouse model, by reducing the overactivation of mTOR pathway [19]. Of note, hyperactive mTOR-mediated signaling in some cases of syndromic ASD [20]. Also, hypomethylation at Raptor introns which is an important component in mTOR signaling pathway in aged sperm were both identified in the present data (Supplementary Figure 3C) and noted in an earlier report [15]. Thus, mTOR inhibitor Rapamicin and/or systemic Igf2 treatments are valuable pharmacotherapies for ASD-like behaviors and aberrant glucose metabolism in the offspring from APA in future studies.

Additionally, it is noteworthy about the hypomethylation of genes, such as Cd38 and Eef2k (Supplementary Figure 3B), associated with the oxytocin signaling pathway [21]. Oxytocin is a widespread neuropeptide existed in various tissues [22, 23]. Generally, oxytocin and activation of the oxytocin signaling pathway in the nervous system are beneficial to social cognition and behaviors. Growing evidence has shown that oxytocin is a potential therapeutic resource for the social core symptoms of ASD [24, 25]. Inactivation of the oxytocin signaling pathway is associated with social impairment, such as ASD, schizophrenia and anxiety disorders [26, 27]. For instance, depletion of Cd38 in mice leads to severe social deficits [28]. However, some reports have suggested that the relatively high expression level of oxytocin in plasma is positively correlated with the severity of social anxiety symptoms [29]. On the other hand, hypomethylation in promoter of OXTR (oxytocin receptor) is a risk factor for increasing amygdala activity towards social anxiety-related words in social anxiety disorder diagnosis (SAD) and increasing cortisol release during the Trier Social Stress Test (TSST) [30]. Moreover, the oxytocin and oxytocin signaling pathway are possibly associated with sexual dimorphism in social cognition and behaviors [31, 32]. Additional studies are necessary to explore whether the epigenetic alterations of oxytocin signaling related genes in aged sperm contribute to the anxious behavior exhibited in open field test and a sexually dimorphic development in F1 and F2 offspring from OF mice. Furthermore, the hypomethylation of TNF signaling related genes in aged sperm have suggested that the chronic inflammatory may happen in the next generation from APA mice. Indeed, it was shown that CD44^high^-expressing activated/memory T cells are induced in offspring of APA mice [15]. Chronic and acute inflammatory changes, especially elevation of TNFα levels in animal models and autistic children, are tightly associated with ASD symptoms [33, 34].

In conclusion, current findings not only confirm the risk of ASD-like behaviors and aberrant glucose metabolism in F1 and F2 offspring from aging mouse models, the data reported here also provide a valuable source of information based on WGBS data in aged sperm. These findings should be of interest to investigators in order to elucidate the underlying mechanism(s) mediating the intergenerational and transgenerational transmissions.

## MATERIALS AND METHODS

### Animals

The 6-week- and 6-month-old male C57BL/6J mice (founder mice) were purchased from SLAC Laboratory Animal Co., Ltd (Shanghai, China) and bred in SPF class house under light:dark cycle of 12hr:12hr, and temperature (21±1^o^C) until the ages of these mice reached to 2 months, 12 months or 18 months before experiments began. To obtain the F1 offspring, the health male mice, grouped into young father (YF-F0, n=15), old father (OF-F0, n=15) and very old father mice (VOF-F0, n=15), were bred with three virgin 2-month-old female mice. Successful mating was confirmed by the presence of sperm plugs. To obtain the F2 generation, the cross-breeding scheme was performed for the 5 groups as follows: 1) YF-mF1 x YF-fF1, 2) OF-mF1 x YF-fF1, 3) VOF-mF1 x YF-fF1, 4) OF-mF1 x OF-fF1 and 5) VOF-mF1 x VOF-fF1. Cross-breeding design is shown in Fig1 A. The female mice with vaginal plugs were removed and singly housed to allow the completion of gestation and to breed offspring. The female mice without plugs were also removed and replaced by other virgin female mice in corresponding cages. The fertility of founder mice was determined by sperm motility, virginal plug occurrence and litter size.

### Ethics statement

All experiments using animals were conducted in accordance with the guidelines of the Animal Care and Use Committee of the Shanghai Jiao Tong University, School of Medicine. These animal experiments were approved by the Jiao Tong University, School of Medicine animal ethics committee (approval No. GKLW2016-31).

### Litter size, body weight and sexual ratio in F1 and F2

The day of delivery was designated postnatal day 0 (P0) and the pups were weighted at P0, 3 weeks, 4 weeks, 6 weeks and 8 weeks. The litter size and sexual ratio of F1 and F2 generations were recorded at 1 week. After weaning on postnatal day 21, males and females were housed separately and tested for metabolic and behavioral changes at specified time points used established protocols.

### Glucose and lipid metabolism assays for the founder mice and F1, F2 offspring

The glucose tolerance test was conducted according to previous studies for founder mice, F1 and F2 offspring at 6 and 8 weeks of age [18]. Briefly, the mice were fasted overnight with free access to water *ad libitum*. Fasting blood glucose levels in blood samples from the tail vein were determined by a hand-held glucometer (ONETOUCH UltraEasy, Johnson) the next morning. Thereafter, these mice were injected with glucose (2 g/kg body weight in PBS) intraperitoneally and blood samples were collected from the tail vein at 0, 15, 30, 60 and 120 minutes. The insulin concentrations and plasma triglyceride (TG) levels were determined using the Mouse C-peptide ELISA Kit (ml001995, Mlbio, Shanghai, China) and a relevant kit (ml076636, Mlbio, Shanghai, China), respectively, according to the protocols provided by the manufacturers. Briefly, the plasma from mice was mixed with the reagents in the kits and the absorbance at 450nm for C-peptide and 420nm for TG was detected using a luminometer (Synergy h1 hybrid microplate reader, BioTek, USA). All animals were kept in their original cages with free access to food and water.

### Adult behavioral experiments

The founder mice, F1 and F2 offspring were tested in behavioral experiments. Prior to the beginning of all behavioral experiments, the mice were introduced into the testing room for 1 h to habit the environment, and were returned to their original cages after testing. The apparatus used in the studies was washed with 70% ethanol and water sequentially between subjects. All behavioral experiments were performed blindly, and the behavior of mice was recorded by a Noldus video analysis system (Noldus Information Technology, Wageningen, The Netherlands) for further detailed analysis by computers and manual work using established procedures [35, 36].

### Open field test

The mice were placed into the center of an arena (40-cm width x 40-cm length x 40-cm height) and tracked position by a camera for 20 min to detect locomotor activity and anxiety-related behavior. The locomotor activity was determined by the number of lines crossed by the mouse during the entire test, while the extent of anxiety was determined by the cumulative time spent in the central area by established procedures as described [37, 38].

### Elevated plus maze

Mice were placed on the central platform, facing one of the closed arms and permitted to explore for 5 min freely on the apparatus, which was elevated 45 cm above the floor. The behavior of the animals was recorded by an overhead camera. The cumulative time spent in the open arms was computed to assess the exploration activity and the extent of anxiety by established protocol [39].

### Marble burying test

Firstly, we prepared the testing cages (30 cm x18 cm x 15 cm) filled with corn bedding material to a 3-4-cm depth. In the habituation trial, mice were placed individually in the testing cages for 20 min, and then returned to their home cages thereafter. Next, we placed twenty glass marbles at 3-cm intervals on the surface of the bedding material in the habituated cages. In the test trial, the mice were replaced in the habituated test cage for 20 min. At the end of the experiments, the mice were returned to their home cages, and the number of buried marbles covered more than 50% by bedding was recorded manually in each testing cage as described [39].

### Three-chamber social approach

The apparatus is an opaque plastic box (20 x 15 x 25 cm) divided by transparent plastic walls into three chambers. The dividing walls have an opening (10 cm) in the center to permit mice to explore freely. In the habituation stage, the test mice were placed in the center chamber and freely explored for 20 min in the apparatus. Then, the test mouse was moved into a clean cage while waiting for the testing stage. For the test session, an unfamiliar C57 male mouse as a stranger was placed in a small wire cylinder (8 cm diameter x 15.5 cm height) in one of the side chambers. The empty identical cylinder as a non-social object was placed in the opposite chamber. Next, the test mouse was placed in the center chamber again and allowed to freely explore the apparatus for 10 min. The movement of mice and the time spent in each chamber were measured using a Noldus system as described [40].

### Sperm preparation

The cauda epididymis was dissected and cut from euthanasic mice. Next, the tissues were cut into small pieces and incubated in 500 ul of BWW solution (G2585, Solarbio, Beijing, China) at 37o in a humidified 5% CO_2_ atmosphere for 15 min to free sperm from the epididymis. A small quantity of sperm was analyzed by CASA assay to examine the parameter of sperm motility. The other sperm were precipitated by centrifugation at 500 g for 5 min at room temperature for the DNA methylation sequencing assay.

### Whole-genome bisulfite sequencing (WGBS) mapping and relative analysis

Genomic sperm DNA was extracted using the Easy Pure Genomic DNA Kit (EE101, Transgen Biotech, Beijing, China) for construction of WGBS libraries. In brief, DNA was bisulfite converted with the EZ DNA Methylation-Gold™ Kit (Zymo Research) which was then used WGBS library construction according to the protocol of the Swift Biosciences Accel-NGS Methyl-Seq DNA Library kit (NovelBio Corp. Laboratory, Shanghai). The tagged cDNA libraries were used for 150 bp paired-end sequencing in a single lane of the Illumina XTEN with 10%-15% phi-X for base balance. Before reads mapping, clean reads were obtained from the raw reads by removing the adaptor sequences utilizing Trim Galore using the following parameters: (i) Quality:2 0; (ii) minLength: 50; (iii) TrimN: True; and (iv) Max Error Rate: 0.1. The clean reads were then aligned to mouse genome (Version: mmu10.NCBI.p4) using the Bismark program. MethylKit, an R package for DNA methylation analysis [41], was applied for BS Statistics calculation and differentially methylated region analysis. Significant differential methylation regions (DMRs) were filtered following the criteria at: DMR > 0.2; q-value < 0.05 and annotated by ChIP seeker. DMRs in promoter regions (up to 3 kb upstream) were divided into ’hypermethylated’ or ‘hypomentylated’ in the aged sperm versus young sperm samples. Genes overlapped with DMRs in whole genome were further analyzed by GO analysis and pathway analysis, according to the Gene Ontology which is the Wey functional classification of NCBI and KEGG (Kyoto Encyclopedia of Genes and Genomes) website (http://www.genome,jp/Kegg/).

### Statistical analysis

Statistical analysis was performed by using GraphPad Prism5. Statistical significance was determined by unpaired, two-tailed t-test for three chamber assay, linear regression analysis for all GTT, ITT and insulin concentration examines and one-way ANOVA for other assays. NS (not significant), *, **, and ***, represent *P*>0.05, *P*≤0.05, *P*≤0.01 and *P*≤0.001, respectively.

## Supplementary Material

Supplementary Figures

Supplementary Table 1

Supplementary Table 2

Supplementary Table 3

Supplementary Table 4
