# Supplementary material for: Autism-like behaviors and abnormality of glucose metabolism in offspring derived from aging males with epigenetically modified sperm
Source: Aging (Albany NY). 2020 Oct 13;12(19):19766–84. doi: 10.18632/aging.104061 (PMC7732289; doi:10.18632/aging.104061)
Supplement: Supplementary Figures [file aging-12-104061-s001..pdf]

SUPPLEMENTARY FIGURES

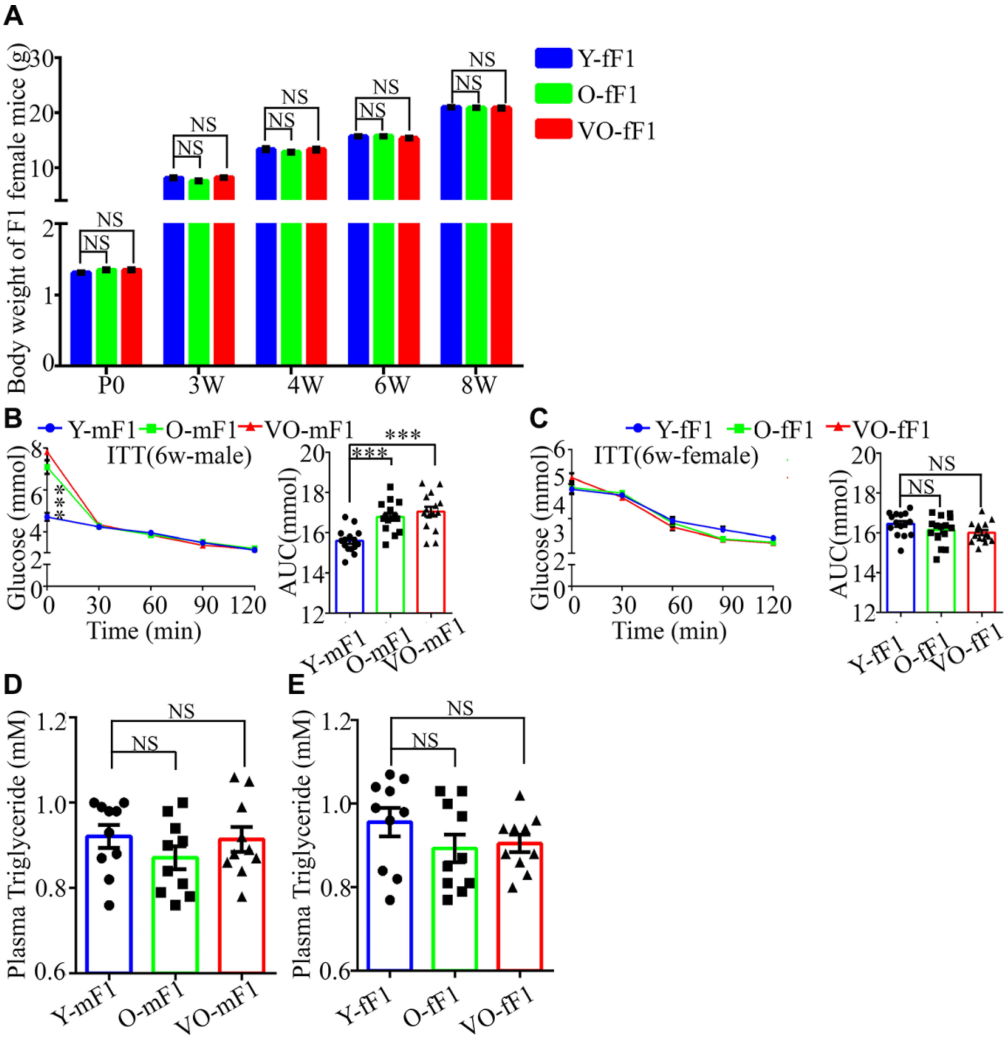

Supplementary Figure 1. The body weight, insulin tolerance test and plasma triglyceride concentrations in OF-F1 and VOF-F1 male and female offspring. (**A**) The body weight trajectories in female F1 offspring (n=30 female offspring for each group, ≥ 5 litter size/group). (**B** and **C**) Blood glucose during an insulin tolerance test (ITT) and AUC results at 6 weeks. (n=15/each sexual offspring, ≥ 5 litter size/group). (**D** and **E**) Plasma triglyceride concentrations in F1 male (**D**) and female (**E**) offspring at 15 weeks. Data are presented as the mean ± s.e.m. NS, P≥0.05, (linear regression analysis for ITT assay and one-way ANOVA for other results).

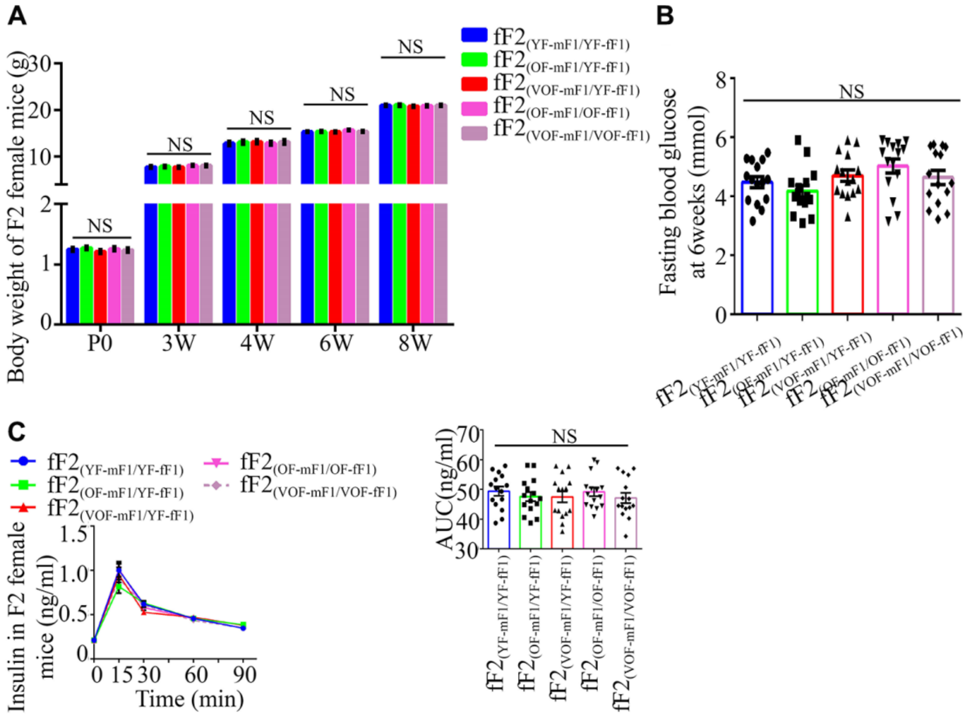

Supplementary Figure 2. The body weight and plasma insulin concentrations in F2 progeny. (**A**) the body weight trajectories in female F2 progeny. (n=30 female offspring for each group, ≥ 5 litter size/group). (**B**) Fasting blood glucose. (n=15 female offspring for each group, ≥ 5 litter size/group) (**C**) Plasma insulin concentration in glucose-stimulated conditions. (n=15/female offspring for each group, ≥ 5 litter size/group). fF2 was referred as to the female progeny from F1 mice, respectively. Data are presented as the mean± s.e.m. NS, P≥0.05 versus control (one-way ANOVA).

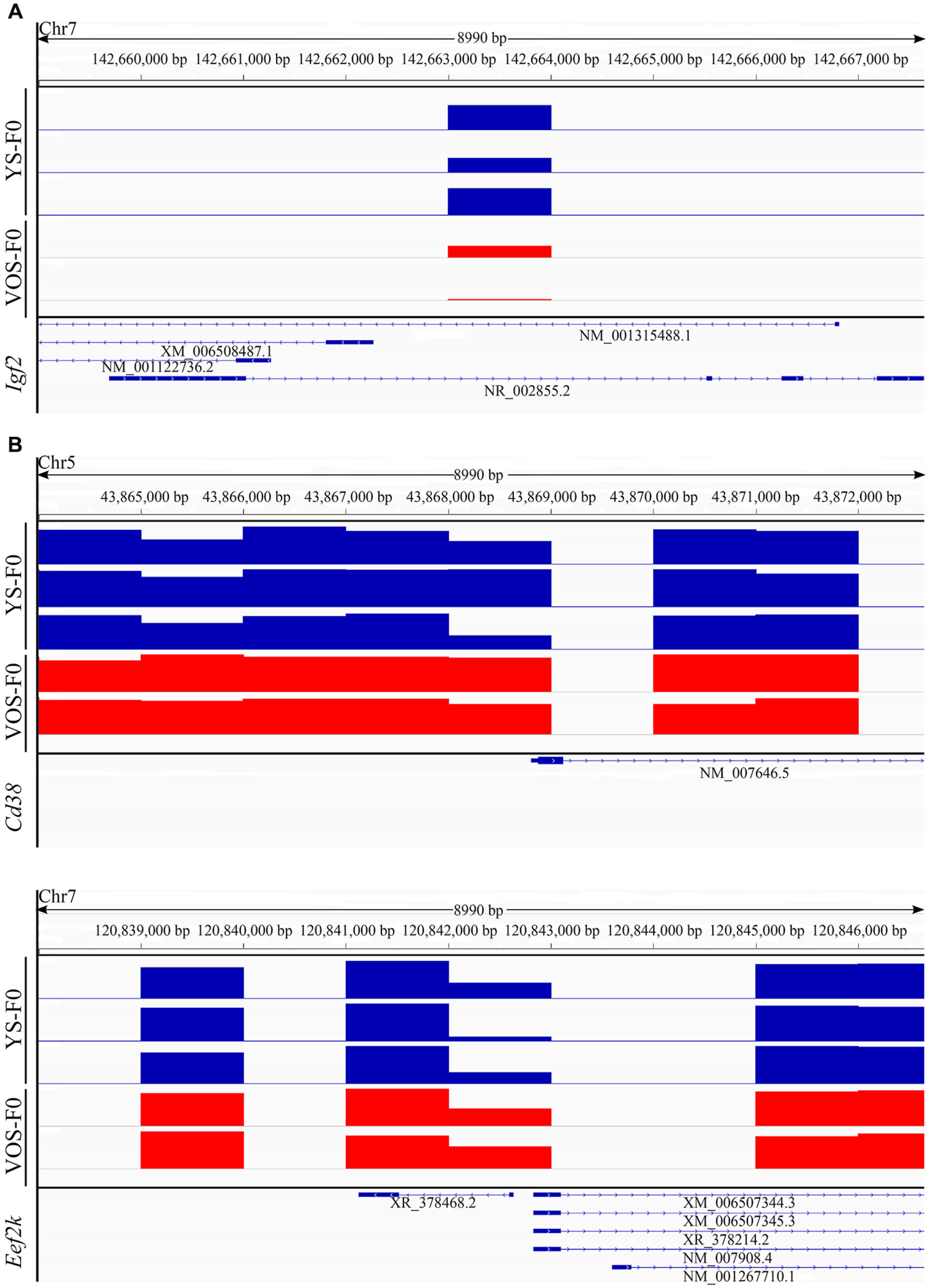
A
Chr7
8990 bp
142,660,000 bp 142,661,000 bp 142,662,000 bp 142,663,000 bp 142,664,000 bp 142,665,000 bp 142,666,000 bp 142,667,000 bp
YS-F0
VOS-F0
Igf2
NM_001315488.1
XM_006508487.1
NM_001122736.2
NR_002855.2
B
Chr5
8990 bp
43,865,000 bp 43,866,000 bp 43,867,000 bp 43,868,000 bp 43,869,000 bp 43,870,000 bp 43,871,000 bp 43,872,000 bp
YS-F0
VOS-F0
Cd38
NM_007646.5
Chr7
8990 bp
120,839,000 bp 120,840,000 bp 120,841,000 bp 120,842,000 bp 120,843,000 bp 120,844,000 bp 120,845,000 bp 120,846,000 bp
YS-F0
VOS-F0
Eef2k
XR_378468.2
XM_006507344.3
XM_006507345.3
XR_378214.2
NM_007908.4
NM_001267710.1

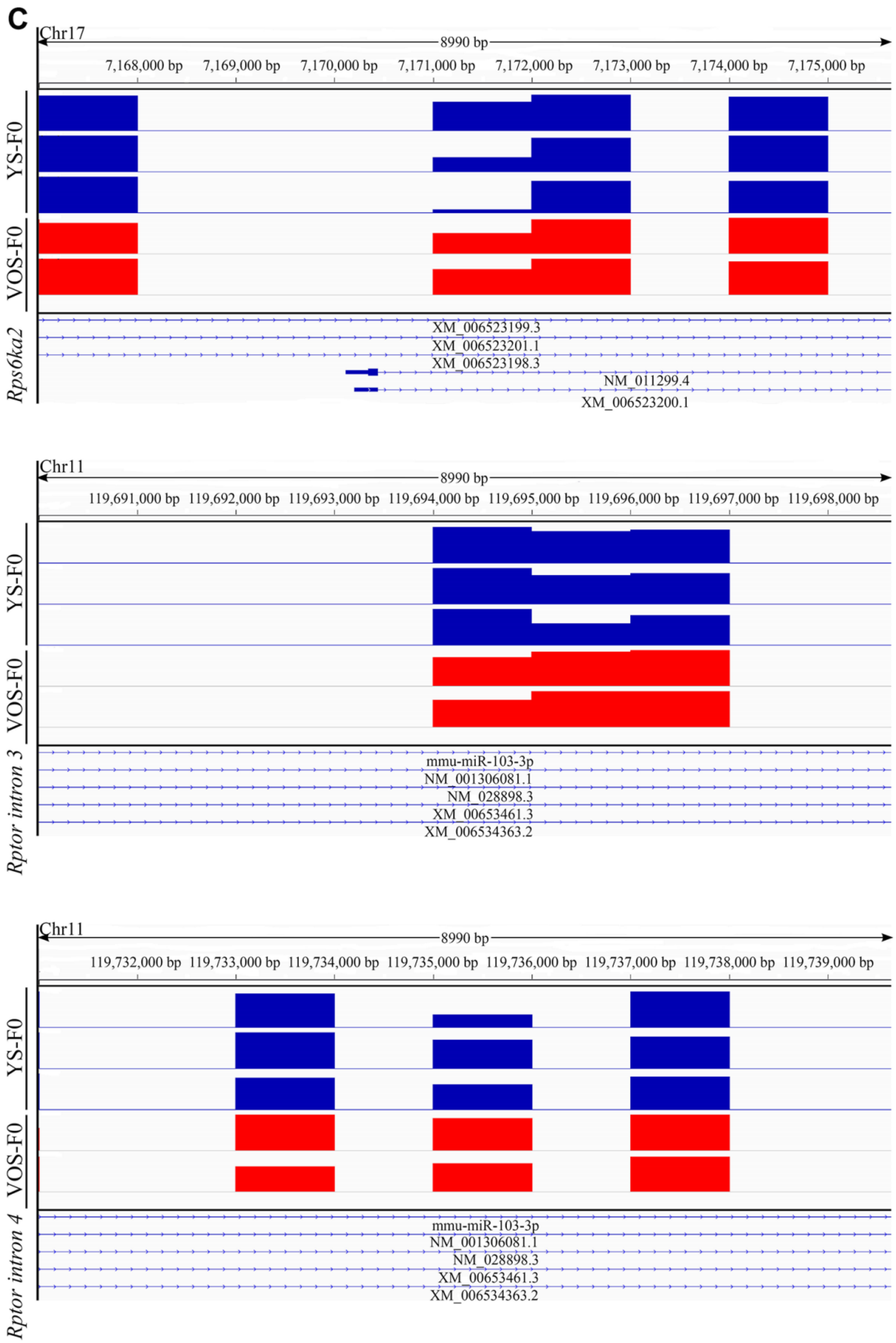

Supplementary Figure 3. The Genome Browser tracks of exemplar DMR of *Igf2* (NM_001315488.1), *Cd38* (NM_007646.5), *Eef2k* (NM_007908.4), *Rps6ka2* (NM_011299.4) and Rptor (NM_00130681.1) genes. (**A**) The Integrative Genomics Viewer (IGV) of the DMR on *Igf2* promoter region. (**B**) The IGV of the DMR on *Cd38* and *Eef2k* promoter regions, which are involved in Oxytocin signaling pathway. (**C**) The IGV of the DMR of *Rps6ka2* promoter region and *Rptor* intron 3 and 4 regions, which are involved in mTOR signaling pathway. The samples named YS-F0 refers as young mice sperm-F0 and VOS-F0 refers as very old mice sperm-F0. The high levels of blue and red strips indicate the DNA methylation levels.
